# High-efficiency SOI-based metalenses at telecommunication wavelengths

**DOI:** 10.1515/nanoph-2022-0480

**Published:** 2022-10-21

**Authors:** Taesu Ryu, Moohyuk Kim, Yongsop Hwang, Myung-Ki Kim, Jin-Kyu Yang

**Affiliations:** Department of Optical Engineering, Kongju National University, Cheonan, 31080, Republic of Korea; KU‐KIST Graduate School of Converging Science and Technology, Korea University, Seoul, 02841, Republic of Korea; Institute of Application and Fusion for Light, Kongju National University, Cheonan, 31080, Republic of Korea; Laser Physics and Photonics Devices Lab, STEM, University of South Australia, Mawson Lakes, SA 5095, Australia

**Keywords:** metalens, metasurface, silicon photonics

## Abstract

We demonstrated silicon-on-insulator (SOI)-based high-efficiency metalenses at telecommunication wavelengths that are integrable with a standard 220 nm-thick silicon photonic chip. A negative electron-beam resist (ma-N) was placed on top of the Si nanodisk, providing vertical symmetry to realize high efficiency. A metasurface with a Si/ma-N disk array was numerically investigated to design a metalens that showed that a Si/ma-N metalens could focus the incident beam six times stronger than a Si metalens without ma-N. Metalenses with a thick ma-N layer have been experimentally demonstrated to focus the beam strongly at the focal point and have a long depth of field at telecommunication wavelengths. A short focal length of 10 μm with a wavelength-scale spot diameter of approximately 2.5 μm was realized at 1530 nm. This miniaturized high-efficiency metalens with a short focal length can provide a platform for ultrasensitive sensors on silicon photonic IC.

## 1 Introduction

Since the concept of metasurfaces was proposed by the Cappasso group in 2011 [1], many applications of metasurfaces have been suggested and demonstrated not only in alternative conventional optics such as lenses [2], [3], [4], [5], [6], [7], diffraction optic elements [8], [9], [10], and waveplates [11], [12], [13], but also in unconventional functional optics such as polarimetric sensors [14], [15], [16], optical vortex converters [17], [18], [19], and abnormal beam steering [20], [21], [22]. In particular, metalenses have attracted the attention of many researchers because of their ultrathin structures and engineerable lens properties in contrast to conventional single convex/concave lenses [3, 23], [24], [25]. Subsequently, more functionalities of metalenses have been developed, including broadband achromatic lenses [3, 4, 6, 7, 26], birefringence lenses [27, 28], and zoom lenses [29], [30], [31].

There are several methods to realize the concept of a metasurface. The first method uses Mie resonance in the nanostructures. Generally, the incident light is scattered at wavelength-scale optical structures, which modulate the amplitude of the scattered light with a phase delay in the propagation direction. The first generation of metasurfaces was demonstrated using localized surface plasmon resonance [1]. The second method to create a metasurface is the so-called Huygens metasurface, which spectrally overlaps the electric dipole (ED) and magnetic dipole (MD) resonances without intensity reduction [32]. The third method for realizing 2*π* phase modulation is the Pancharatnam–Berry (PB) phase or geometric phase [9]. Because the phase of light accumulates owing to the orientation of the anisotropic nanostructure, the amplitude is maintained as a constant. However, a PB-phase metasurface can control the phase of scattered light for a certain circular polarization. An alternative method for phase control with high efficiency was realized using an effective index of meta-atoms [33].

Photonic integrated circuits (PICs) on silicon are well-known in the photonics society owing to their unique advantages in overcoming electric circuits, such as high-speed modulation, direct fiber-to-chip signal delivery, and low power consumption [34]. Therefore, a silicon-on-insulator (SOI)-based metasurface is an important platform in terms of compatibility with photonic chips [35]. Recently, several attempts have been made to integrate metasurfaces on PIC chips at telecom wavelengths, including wavefront shaping, for efficient coupling [36]. However, owing to symmetry breaking along the out-of-slab direction, the ED and MD resonances do not spectrally coincide, resulting in a reduction in metasurface efficiency [32]. In addition, owing to the thin Si slab of a standard SOI wafer, the operation wavelength should be shorter than the telecommunication wavelength [35, 37]. Otherwise, the Si slab should be thick [38].

In this study, we propose a new type of metalens based on a standard SOI wafer, which is a periodically arranged double-layer meta-atom composed of an electron-beam (e-beam) resist and Si disks. This meta-atom is designed to have a low-refractive-index material both on the top and bottom of the Si disk, hereby preserving the structural symmetry for high efficiency. We believe that the symmetry-preserved SOI-based metasurfaces can be a good platform for on-chip high-efficiency metasurfaces for various Si photonic devices.

## 2 Design of the SOI-based metalens

Generally, the thickness of the Si layer of an SOI wafer for a PIC chip is set to 220 nm to operate a single guided mode at the waveguide structures [39]. A method to realize a high-efficiency metasurface with an ultrathin high-index contrast slab is to spectrally overlap the ED and MD resonances not only with the spectral position but also with the strength and the width [40]. It is worth noting that the quality factor (Q-factor) of both the ED and MD resonances should be the same for unity transmission. To satisfy this condition, the structure should be symmetric along the transmission direction, including dielectric conditions. Therefore, in the case of broken vertical symmetry of the meta-atom, the spectral shapes of the ED and MD resonances are slightly different, and the transmission is significantly reduced. In this study, we propose a high-efficiency compact metalens composed of a Si nanodisk with a remaining e-beam resist on top of a buried SiO_2_ box to preserve vertical symmetry.

**Figure 1:**
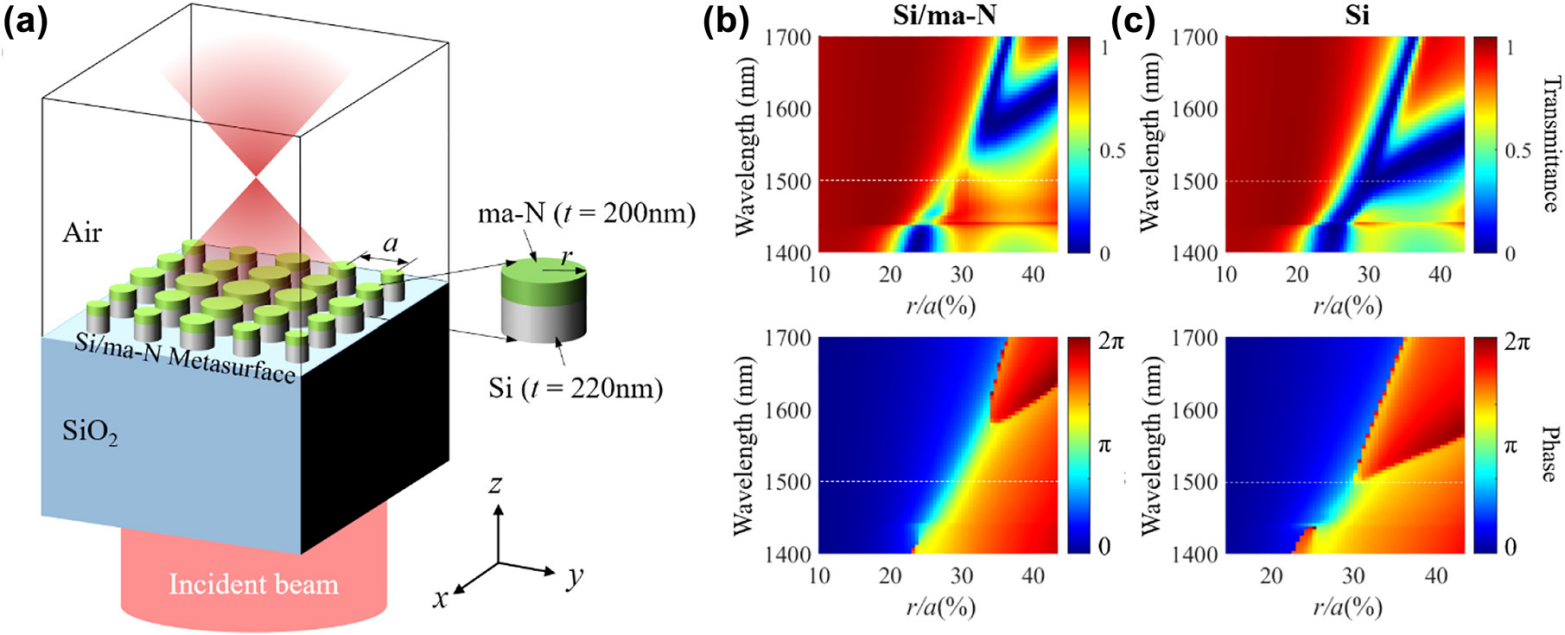
(a) Illustration of the high-efficiency metalens with Si/ma-N disks. Numerically simulated transmittance (top) and phase (bottom) maps of the incident light at (b) Si/ma-N/Air and (c) Si/Air metasurfaces as functions of the wavelength and normalized disk radius. The white dashed lines in (b) and (c) indicate the wavelength at 1500 nm.

Figure 1(a) shows the illustration of the high-efficiency compact metalens with Si/ma-N disk arrays, in which ma-N is a negative-tone e-beam resist (Micro Resist Technology GmbH). To investigate the effect of the ma-N layer on transmittance, we performed numerical calculations of the transmission and phase as functions of the radius (*r*) of the Si/ma-N disk using the three-dimensional finite-difference time-domain (3-D FDTD) method. In the simulations, the thicknesses of the Si and ma-N disks were fixed at 220 and 200 nm, respectively, and the period (*a*) of the Si/ma-N array was fixed at 980 nm, because the spectral widths of both ED and MD resonances become similar when the thickness of the ma-N layer becomes thick as shown in Figures S1 and S2 in the Supplementary Material . The refractive indices of ma-N and SiO_2_ were set as 1.607 and 1.4657, respectively. Figure 1(b) and (c) show the transmittance and phase of the transmitted light as functions of the wavelength and normalized radius *r*/*a* of the arrays of Si/ma-N and Si meta-atoms, respectively. In the case of the Si/ma-N meta-atom, broadband 2*π* phase modulation can be achieved from 1400 to 1600 nm by varying *r*/*a*. In particular, the transmittance was over 70% near 1500 nm, marked with white dashed lines in Figure 1(b) and (c) (see Figure S1). However, for the Si meta-atom without the ma-N layer, the bandwidth of 2*π* phase modulation is narrow (1430–1500 nm), and the transmittance is near zero in the bandwidth. Additional data on the transmittance and phase as functions of the disk radius can be found in Figure S3 in the Supplementary Material. From these results, we expect that Si/ma-N metasurfaces can perform as SOI-based high-efficiency compact metasurfaces, which are compatible with a standard CMOS fabrication process.

**Figure 2:**
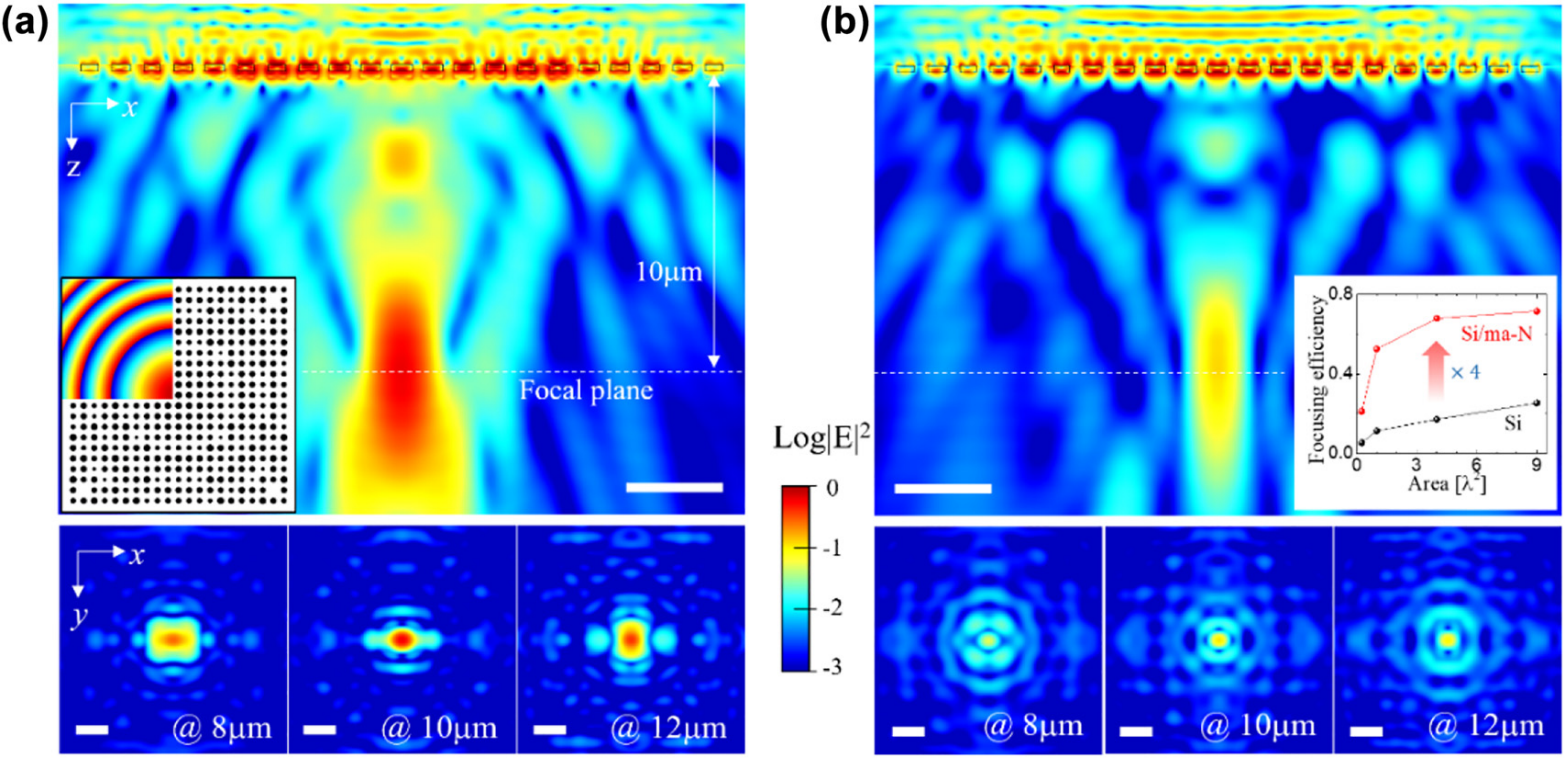
Numerical results of the intensity distribution for metalens with *f* = 10 μm. (a) Si/ma-N disk metalens. (b) Si disk metalens. The upper panels are *xz*-plane views along the optical axis and the lower panels are *xy*-plane views at various *z*-positions. The left inset in (a) shows the designed phase map and corresponding metalens structure. The inset in (b) is the focusing efficiency graph as a function of focusing area. The white bar denotes 3 μm (approximately 2*λ*).

The SOI-based high-efficiency compact metalens was designed with a simple relationship between the required phase and structural parameters extracted from Figure 1(b). For a given focal length *f*, the desired phase profile *ϕ*(*x*, *y*) at the metasurface follows equation [2]:

$$\phi \left(x,y\right)=\frac{2\pi }{\lambda }\left(f-\sqrt{x^{2}+y^{2}+f^{2}}\right)$$

where *λ* is the wavelength in free space. In the simulation, we fixed *λ* at 1500 nm. The left inset in Figure 2(a) shows the calculated phase map for the metalens with *f* = 10 μm and its corresponding structure. Figure 2(a) shows the time-averaged intensity profiles on a logarithmic scale for the Si/ma-N metalens. Based on the *xz*-plane view (upper panel), the numerical result of the focal length is approximately 9.68 μm (error <5%). Based on the *xy*-plane-view intensity profiles (the lower panels), the full width at half maximum (FWHM) of the beam spot at the focal plane (10 μm) is approximately 1.08 μm (0.72*λ*). However, metalens consisting of a Si disk array cannot efficiently focus light on the focal plane, as shown in Figure 2(b). From Figure S2(a) and (b), the peak intensity of the Si/ma-N metalens on the focal plane is six times higher than that of the Si metalens. Conversely, the focal length and FWHM of the beam spot is slightly reduced to 9.42 and 0.93 μm, respectively. As a quantitative measure of the performance of metalens, the focusing efficiency is defined as the ratio of the incident power to the focused power within a square area of the focal plane. The focusing efficiency of the Si/ma-N metalens was four times higher than that of the Si metalens, as shown in the right inset of Figure 2(b). In particular, half of the incident light was focused within an area of λ^2^ by the Si/ma-N metalens. This significant difference between the Si/ma-N and Si metasurfaces indicates that a low-index layer on top of the Si disk is essential for achieving high efficiency.

## 3 Fabrication and experimental results

**Figure 3:**
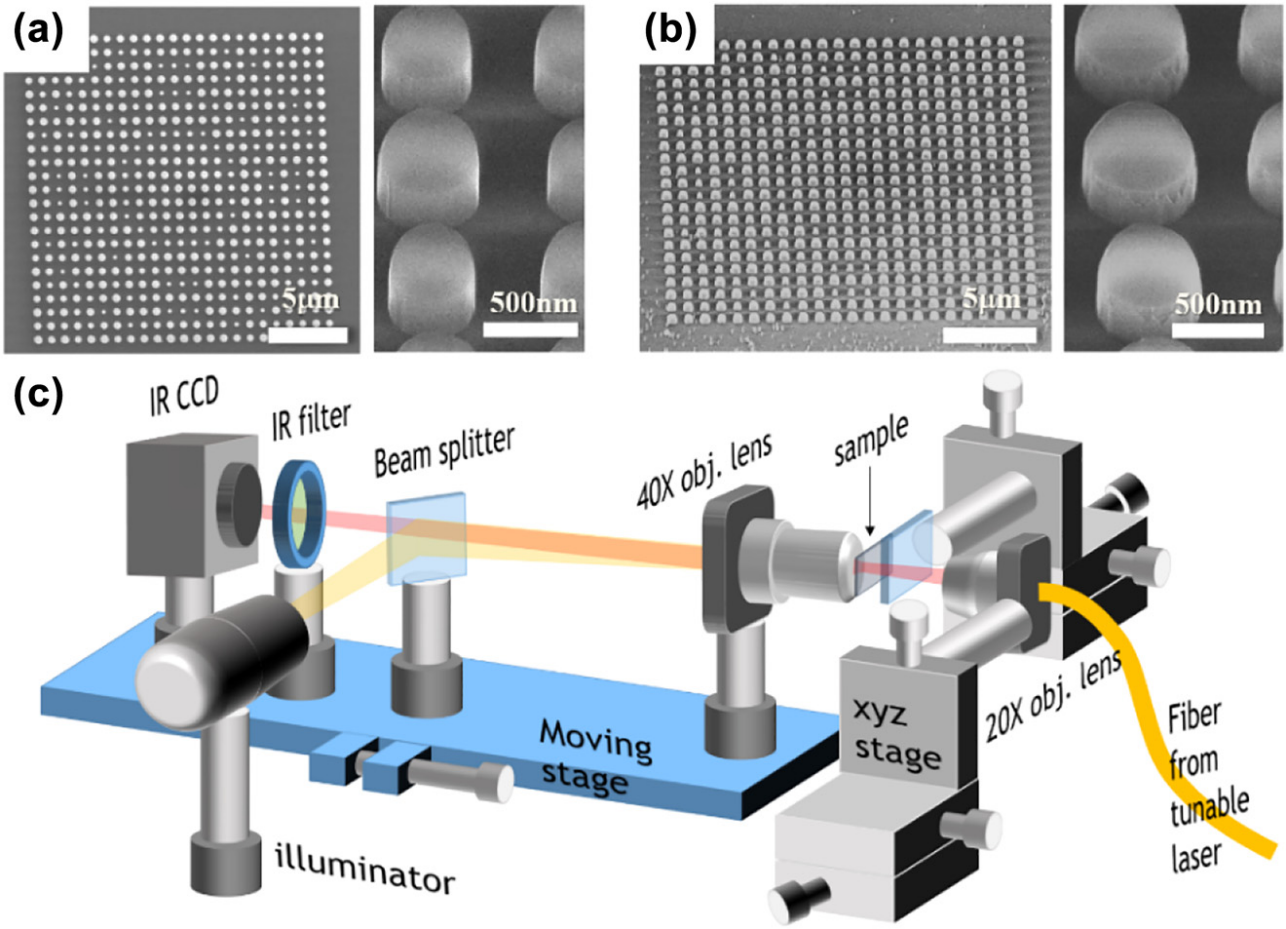
SEM images of the fabricated metalens samples with (a) thick (140 nm) and (b) thin (100 nm) ma-N layers for a 10 μm focal length. (c) Schematic of the experimental setup.

The metalens samples were prepared using a standard SOI wafer composed of a Si slab on top of a 2 μm-thick SiO_2_ box layer. The thickness of the Si slab was 220 nm to support single guided mode in the Si waveguide structures at telecommunication wavelengths. Metalens were fabricated using typical e-beam lithography and a CMOS-compatible etching process. First, the backside of the SOI wafer was polished to reduce light scattering. After completing the SOI wafer cleaning and surface treatment with sulfuric acid hydrogen peroxide mixture (H_2_SO_4_/H_2_O_2_ = 3:1) to improve the surface adhesion with the e-beam resist, a highly sensitive negative-tone e-beam resist film (ma-N 2403, Micro Resist Technology GmbH) was spin-coated on the SOI wafer. The thickness of the coated ma-N was approximately 300 nm. The designed metalens structures were imported into the samples via an e-beam writing process. Subsequently, the e-beam resist was developed with an aqueous-alkaline tetramethylammonium hydroxide (TMAH)-based developer (ma-D 525, Micro Resist Technology GmbH) and post-baked in an oven at 100 °C for 5 min to increase etch resistance and thermal stability. The developed pattern was transferred into the Si slab layer using a fluorine-based (SF_6_ + C_4_F_8_) inductively coupled plasma-reactive ion etching (ICP-RIE) (Multiplex ICP, STS) dry etching process. Finally, metalenses consisting of a Si/ma-N disk array were obtained. To clean the ma-N surface, the ma-N disk layer was etched using an O_2_ plasma etcher (Covance, Femto Science). Figure 3 shows SEM images of the fabricated metalens samples with a 10 μm focal length. The side length of the square metalens pattern is approximately 20 μm, and the thicknesses of the ma-N disk in Figure 3(a) and (b) are approximately 140 and 100 nm, respectively.

The fabricated metalens were characterized using a tunable laser and infrared charge-coupled device (IR CCD) sensor. Figure 3(c) shows the schematic of the experimental setup. First, the fabricated sample with the proper design parameter was found using illuminator and IR CCD, and placed on the optical axis by adjusting the xyz stage of the sample’s mount. The laser light (HP 8168F tunable laser source) delivered by a single-mode fiber was focused on the metasurface using a 20× objective lens (NA = 0.4). The diameter of the incident beam was approximately 20 μm. By moving the stage, the focused beam after metalens was imaged using a 40× aspheric lens (NA = 0.6) and captured by a CCD sensor.

**Figure 4:**
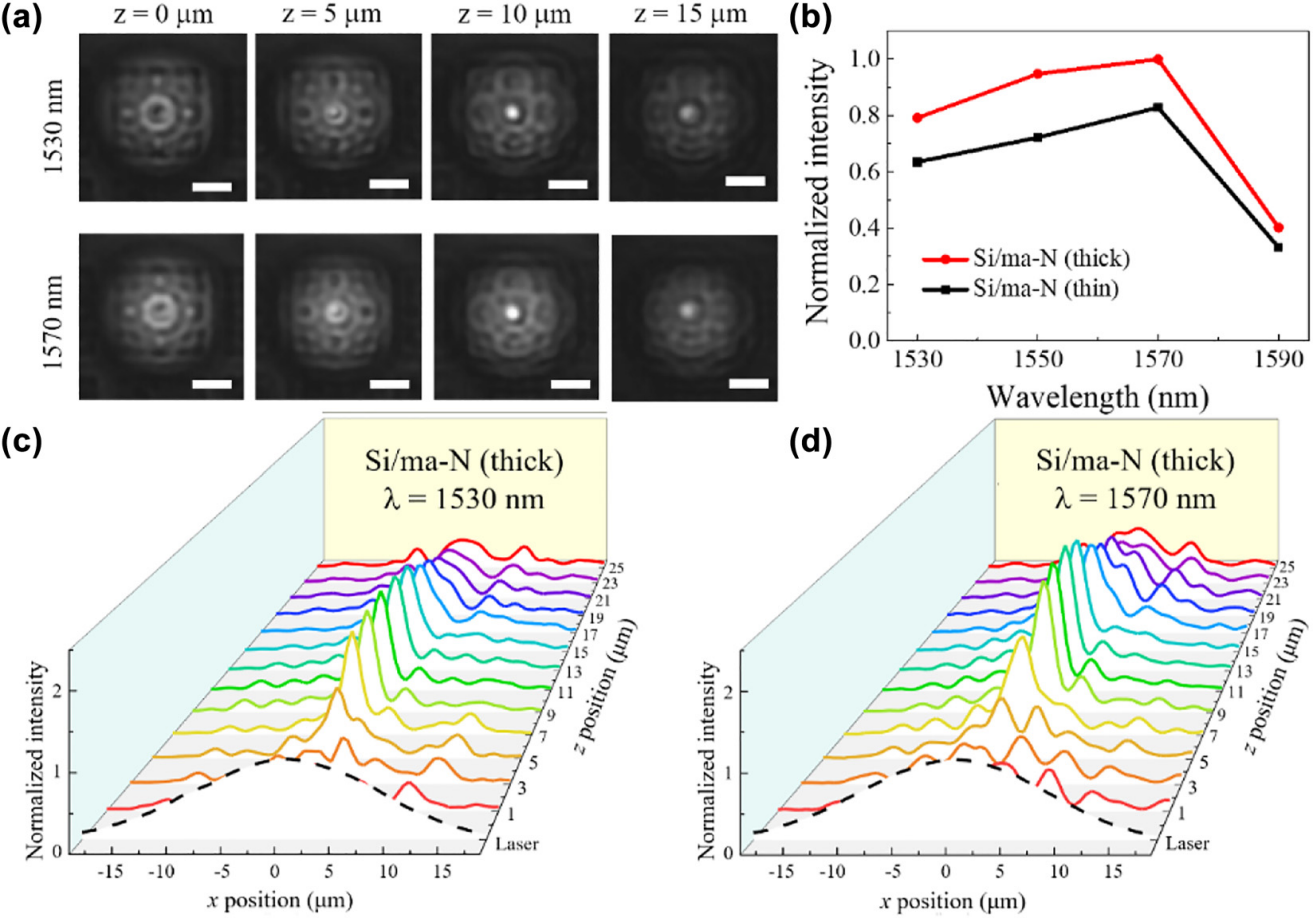
Experimental results of the focused light by metalens. (a) Captured beam images with different distances from metalens. (b) Peak intensity at the focal plane with different wavelengths. (c) Intensity distributions of the transmitted beam by metalens at *λ* = 1530 nm (d) and *λ* = 1570 nm. The white bars in (a) denote a 10 μm length. The dashed lines in (c) and (d) denote the intensity distribution of the incident laser beam at *z* = 0 without metalens.

Figure 4 shows the characteristics of the beam focused by metalens at *f* = 10 μm. Based on the captured image of the beam, as shown in Figure 4(a), it is clear that metalens focused the beam at the designed focal length. From the intensity distribution of the light on the metasurface (*z* = 0 μm), scattered beams at the edge of metalens and a ring-shaped beam at the center were observed (see more details in Movie S1). As the propagation distance (*z*) increased, the ring pattern shrank and finally merged at the focal plane (*z* = 10 μm) as a spot. At *λ* = 1570 nm, metalens still worked well in focusing the beam at the designed focal length; however, the intensity outside the beam spot became slightly bright. From the normalized peak intensity at the focal plane shown in Figure 4(b), the metalens with a thick ma-N layer focuses the light more efficiently by approximately 20% than that with a thin ma-N layer, which could be improved over 20% when the thickness of the ma-N becomes 200 nm (see the Figures S6 and S7 in the Supplementary Material). We also examined the evolution of the propagation beam through metalens for quantitative analysis. Figure 4(c) shows the intensity profiles of the focused beam at *λ* = 1530 nm as the propagation distance changes. The intensity was maximized at the focal position and the FWHM of the beam spot was approximately 2.5 μm. As the wavelength increased, the ripples in the intensity profiles became large, as shown in Figure 4(d). This indicates that the metalens becomes less efficient over the target wavelength of 1500 nm as shown in Figure S5 and Table S1 in the Supplementary Material. However, metalens still worked well in terms of focusing performance.

**Movie S1:** 

## 4 Discussions

**Figure 5:**
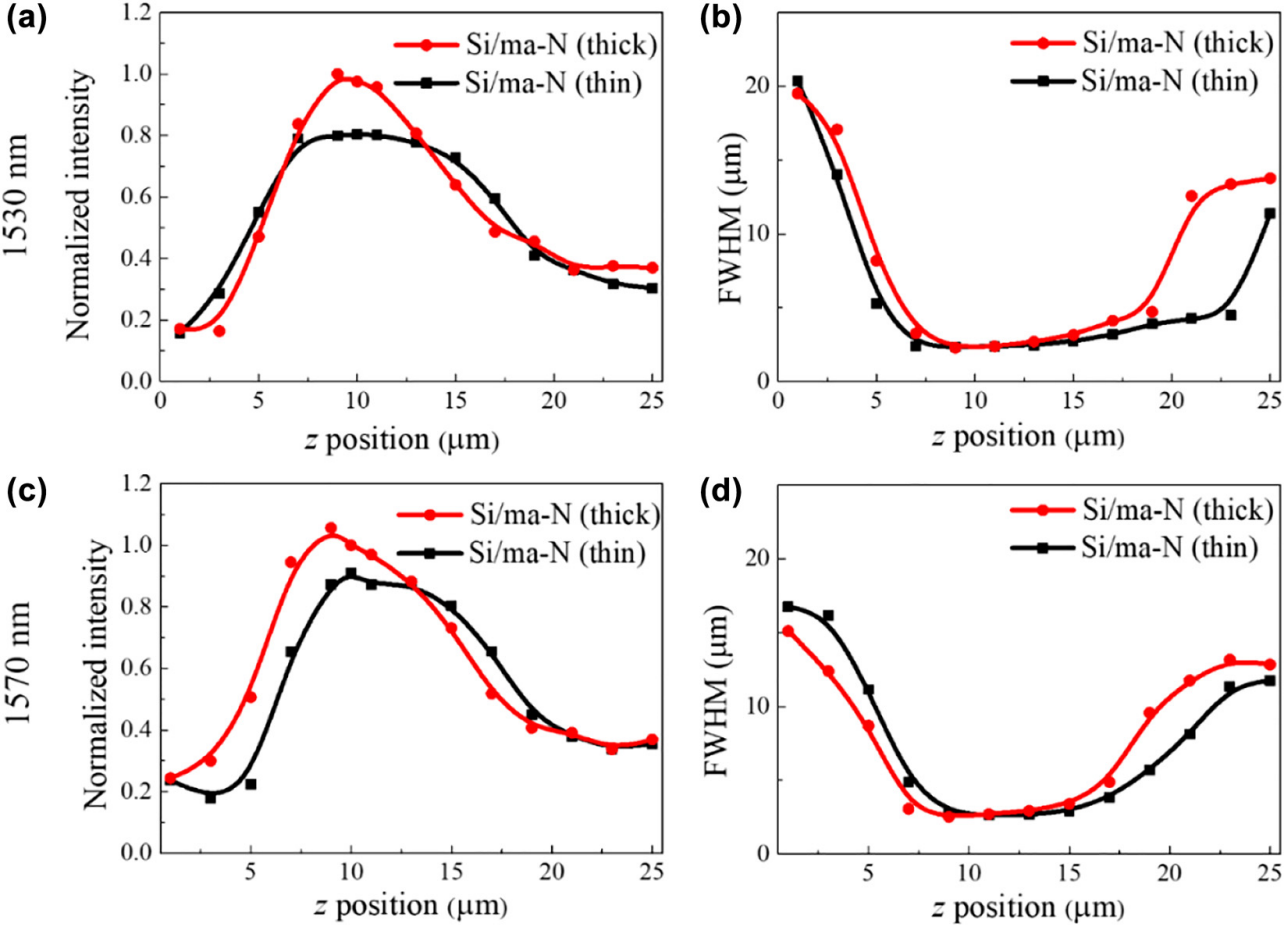
Characteristics of the thin (100 nm) and thick (140 nm) ma-N metalenses with a 10 μm focal length. (a) Normalized peak intensity and (b) FWHM of the propagated beam at *λ* = 1530 nm. (c) Normalized peak intensity and (d) FWHM of the beam intensity at *λ* = 1570 nm.

To quantitatively understand the performance of metalens, the peak intensities and FWHMs of the propagated beam were extracted from the intensity distributions of the transmitted beam. Based on the normalized peak intensity distributions with the *z*-position, as shown in Figure 5(a), the focal length of both the thick and thin ma-N metalenses is 10 μm at *λ* = 1530 nm, which matches well with the designed value. However, the focal length at *λ* = 1570 nm was slightly shifted to 9 and 12 μm for the thick and thin Si/ma-N metalenses, respectively, owing to the wavelength mismatch with the target wavelength. In addition, the focusing efficiency of the thick Si/ma-N metalens was approximately 20% higher than that of the thin metalens at both *λ* = 1530 and *λ* = 1570 nm. Based on the FWHM as a function of the *z*-position shown in Figure 5(b), the beam diameters of the transmitted beam with *λ* = 1530 nm are approximately 3.9 and 4.0 μm for the thick and thin Si/ma-N metalenses, respectively. However, the beam diameters with *λ* = 1570 nm slightly increased to 4.29 and 4.74 μm for the thick and thin Si/ma-N metalenses, respectively. In contrast, the depth of field (DOF) of the thin Si/ma-N metalens at *λ* = 1530 and *λ* = 1570 nm were approximately 45 and 15% longer than that of the thick Si/ma-N metalens, respectively. Considering the small difference (<10%) between the beam spot sizes of the thick and thin metalenses, this large difference in DOF is caused by the phase mismatch owing to the thickness difference of the ma-N layer. Hence, we conclude that the thickness of the ma-N layer is important for creating efficient metalenses with a long DOF and small beam spot.

SOI-based metalenses at telecommunication wavelengths can be good candidates for ultrasensitive sensor platforms. In this study, we numerically investigated the possibility of a highly sensitive surface plasmon resonance (SPR) sensor composed of an SOI-based metalens with a single bowtie air hole in an Au thin film. The center of the bowtie-shaped air hole in the Au thin film was placed at the focal spot of the Si/ma-N metalens, as shown in Figure S3 in the Supplementary Material. Because the incident light is collected by the bowtie-hole nanoantenna by the surface plasmon effect and transmitted through it, the intensity at the Au nanogap could be significantly enhanced [41]. Based on the numerical simulation, the maximum field intensity at the bowtie hole with metalens of *f* = 3 μm can be enhanced twelvefold compared with the bowtie hole without metalens, when the bowtie hole lies on the focal plane.

## 5 Conclusions

We demonstrated high-efficiency SOI-based metalenses with a short focal length at telecommunication wavelengths that are integrable with conventional silicon photonic chips. To increase the transmittance, a negative e-beam resist (ma-N) was placed on top of a Si meta-atom to preserve the vertical symmetry. A compact metalens was designed and numerically investigated using the 3-D FDTD method. The metasurface composed of Si/ma-N disks can achieve broadband phase modulation by changing the radius of the disk, and the Si/ma-N metalens can focus light six times stronger than the Si metalens. We experimentally demonstrated that the Si/ma-N metalens can focus the incident beam at the focal plane with a spot size approximately equal to the wavelength. In particular, a thicker Si/ma-N metalens focuses the light more efficiently at the focal point and has a longer DOF than the thin Si/ma-N metalens. We believe that SOI-based metalenses can provide a good platform for ultrasensitive sensors on silicon photonic IC.

## Supplementary Material

Supplementary Material Details
