# Supplementary material for: High-efficiency SOI-based metalenses at telecommunication wavelengths
Source: Nanophotonics. 2022 Oct 21;11(21):4697–704. doi: 10.1515/nanoph-2022-0480 (PMC11501833; doi:10.1515/nanoph-2022-0480)
Supplement: Supplementary file 3 — Supplementary Material Details [file j_nanoph-2022-0480_suppl.docx]

Supplementary Information:
*High efficiency SOI-based metalenses at telecommunication wavelengths*

Taesu Ryu^1^, Moohyuk Kim^2^, Yongsop Hwang^3,4^, Myung-Ki Kim^2,*^, and Jin-Kyu Yang^1,3, †^

^1^Department of Optical Engineering, Kongju National University, Cheonan 31080, Republic of Korea

^2^KU‐KIST Graduate School of Converging Science and Technology, Korea University, Seoul, 02841 Republic of Korea

^3^Institute of Application and Fusion for Light, Kongju National University, Cheonan 31080, Republic of Korea

^4^Laser Physics and Photonics Devices Lab, STEM, University of South Australia, Mawson Lakes, SA 5095, Australia

[*rokmk@korea.ac.kr](mailto:*rokmk@korea.ac.kr); ^†^jinkyuyang@kongju.ac.kr


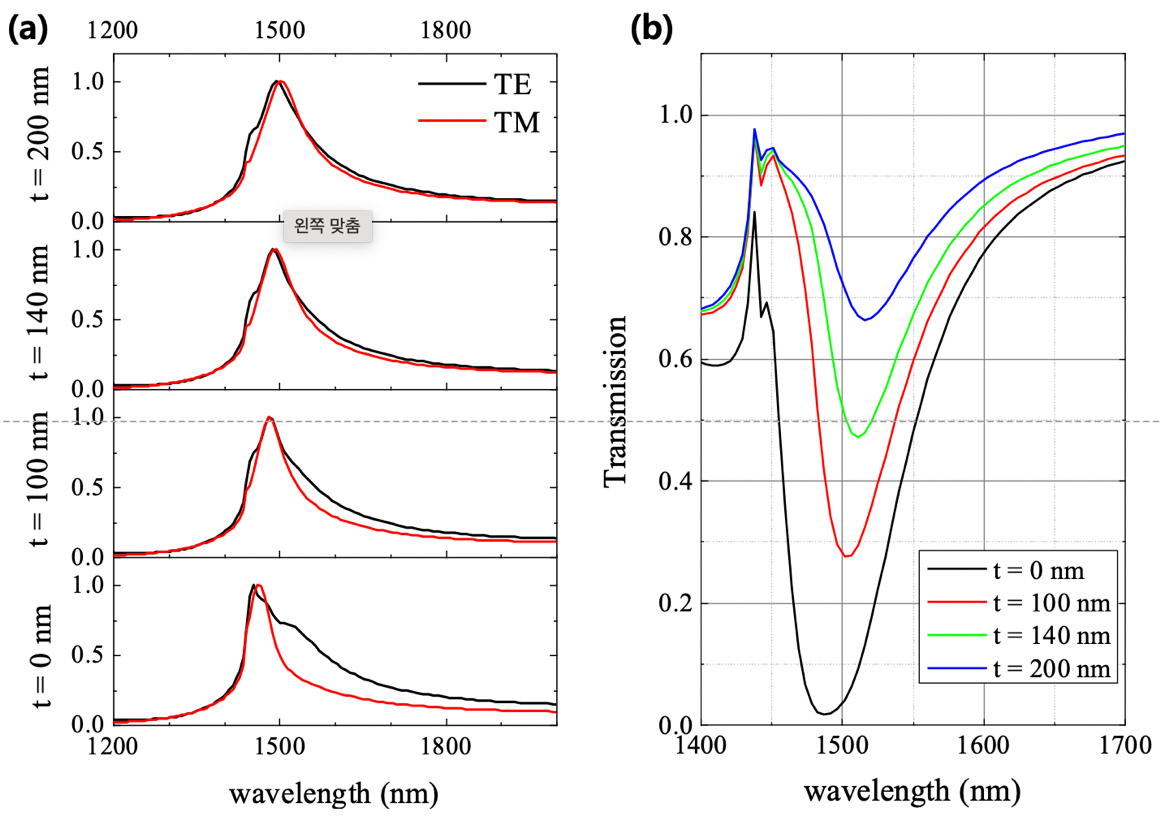


Fig. S1 (a) Spectral responses and (b) transmission spectra of Si/ma-N metasurfaces at different thickness of the ma-N layer at *r/a* = 0.29.


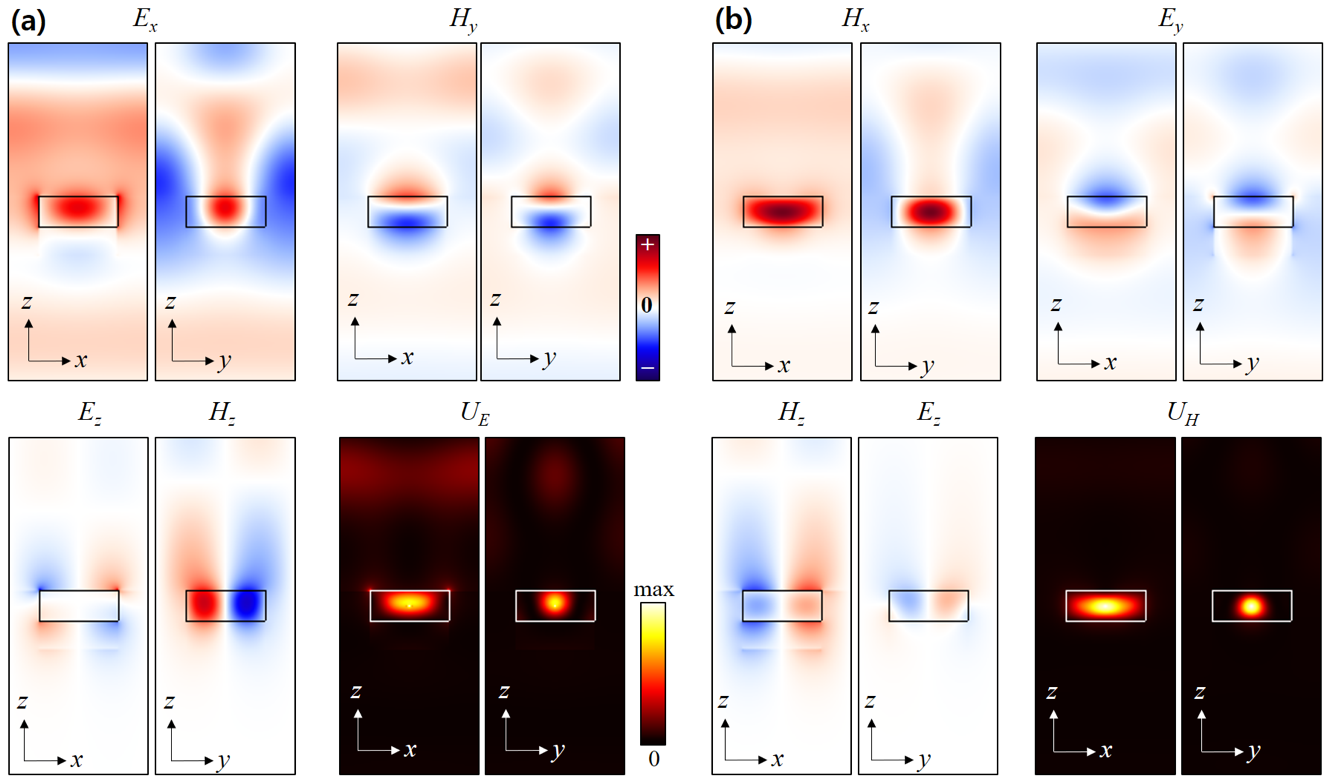


Fig. S2 Electromagnetic field profiles and energy distributions of (a) the ED resonance and (b) MD resonance at λ = 1500 nm in Si/ma-N nanodisk array with *r/a* = 0.29. The thickness of ma-N is 200 nm.


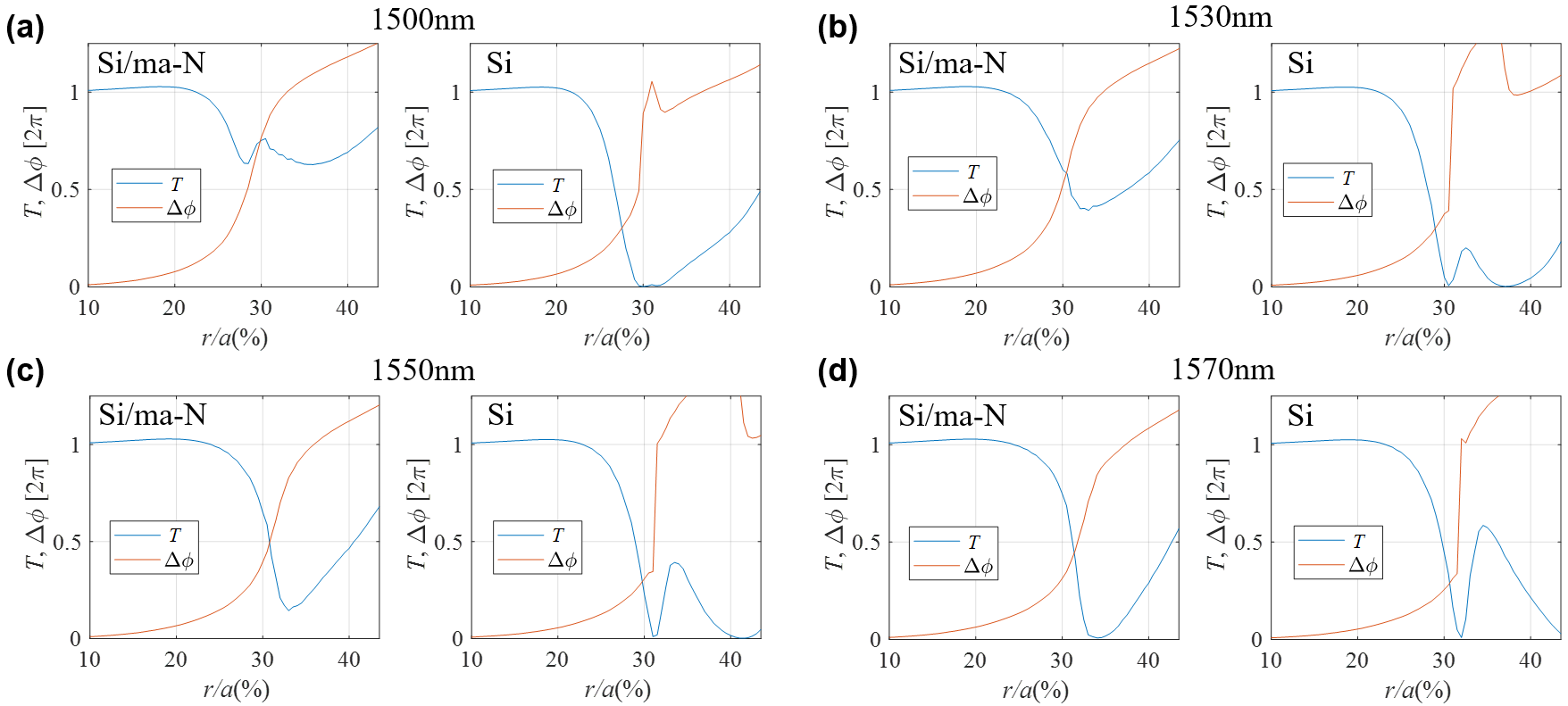


Fig. S3: Numerical results of the transmittance and phase difference of metasurfaces at a given wavelength as a function of meta-atom size. (a) *λ* = 1500 nm, (b) *λ* = 1530 nm, (c) *λ* = 1550 nm, and (d) *λ* = 1570 nm. The left and right graphs correspond to Si/ma-N and Si metasurfaces, respectively.


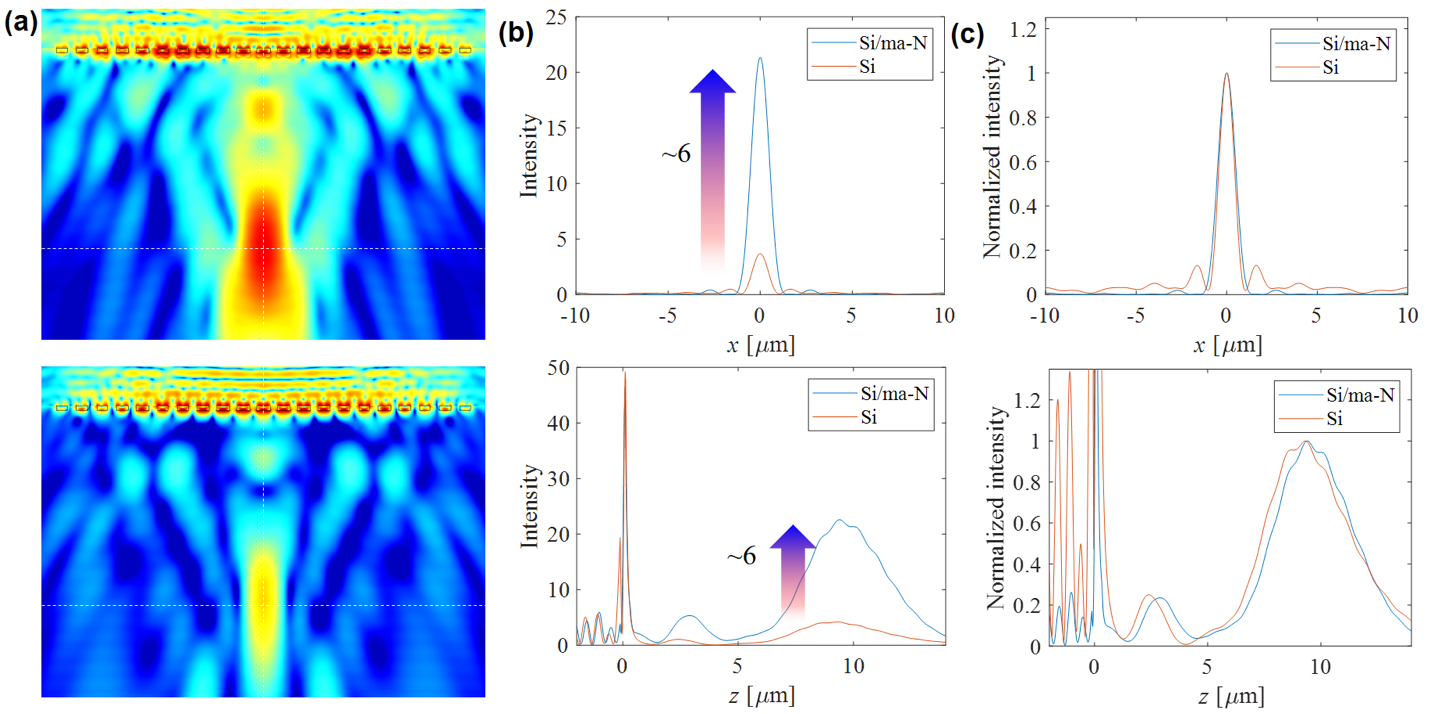


Fig. S4: Numerical results of the characteristics of the Si/ma-N and Si metalenses with *f* = 10 μm. (a) Time-averaged intensity distribution of the focusing beam by the Si/ma-N (upper) and Si (lower) metalenses on a logarithmic scale. (b) Intensity distribution at the focal plane (upper) and along the *z*-position (lower), indicated by the white lines in (a). (c) Normalized intensity distribution at the focal plane (upper) and along the *z*-position.


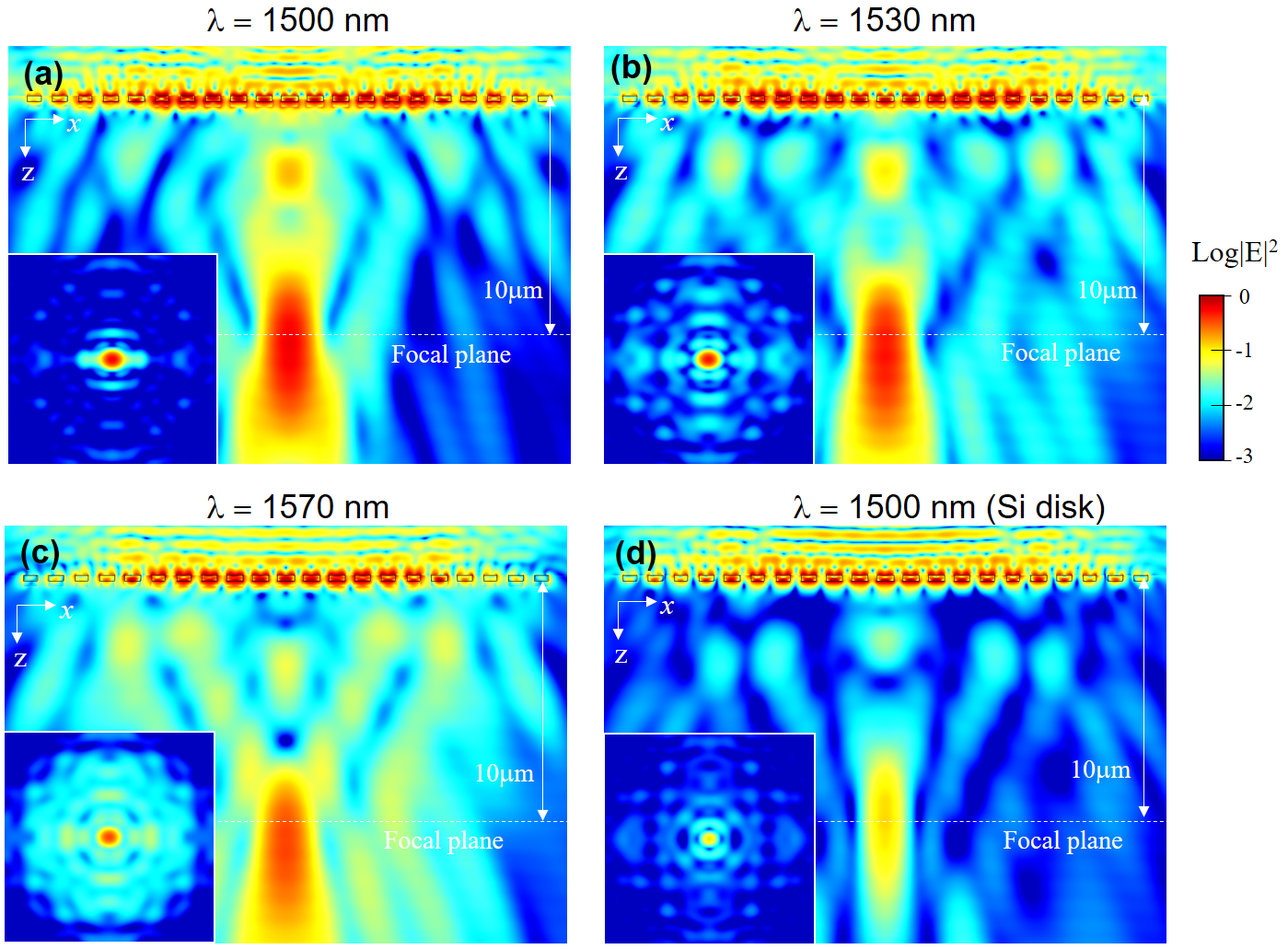


Fig. S5 XZ cut-view of the intensity distribution for the metalens with *f* = 10 μm (a) at 1500 nm, (b) at 1530 nm, (c) at 1570 nm, and (d) at 1500 nm without the ma-N layer. All the images are shown as a logarithmic scale and the inset images are the intensity distribution in the focal plane.

Table S1 Calculated focused power and FWHM of the focusing spot at different wavelengths.

| *Wavelength* | *1500 nm* | *1530 nm* | *1570 nm* | *1500 nm* (*Si*) |
| --- | --- | --- | --- | --- |
| *Focused power* (*within λ^2^*) | 9.72e9 (100%) | 7.80e9 (80%) | 7.13e9 (73%) | 1.54e9 (16%) |
| *FWHM* | 1.08 μm | 1.04 μm | 1.16 μm | 0.96 μm |


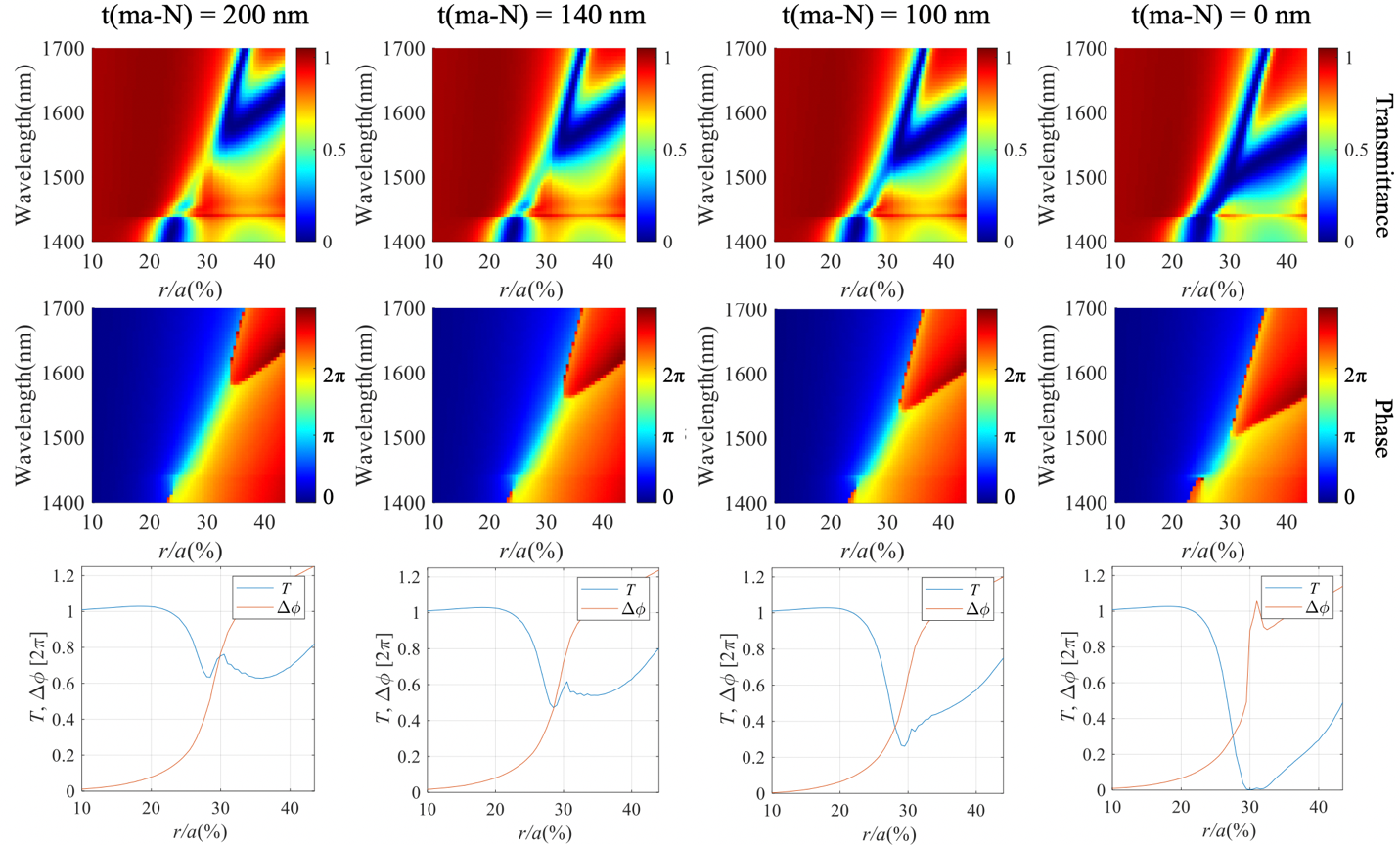


Fig. S6 Numerically simulated transmission (top) and phase (middle) maps of the incident light as functions of the wavelength and disk radius, and transmittance and phase difference at λ = 1500 nm (bottom). From the left to the right column, the thickness of ma-N layer changes from 200 nm to 0 nm.


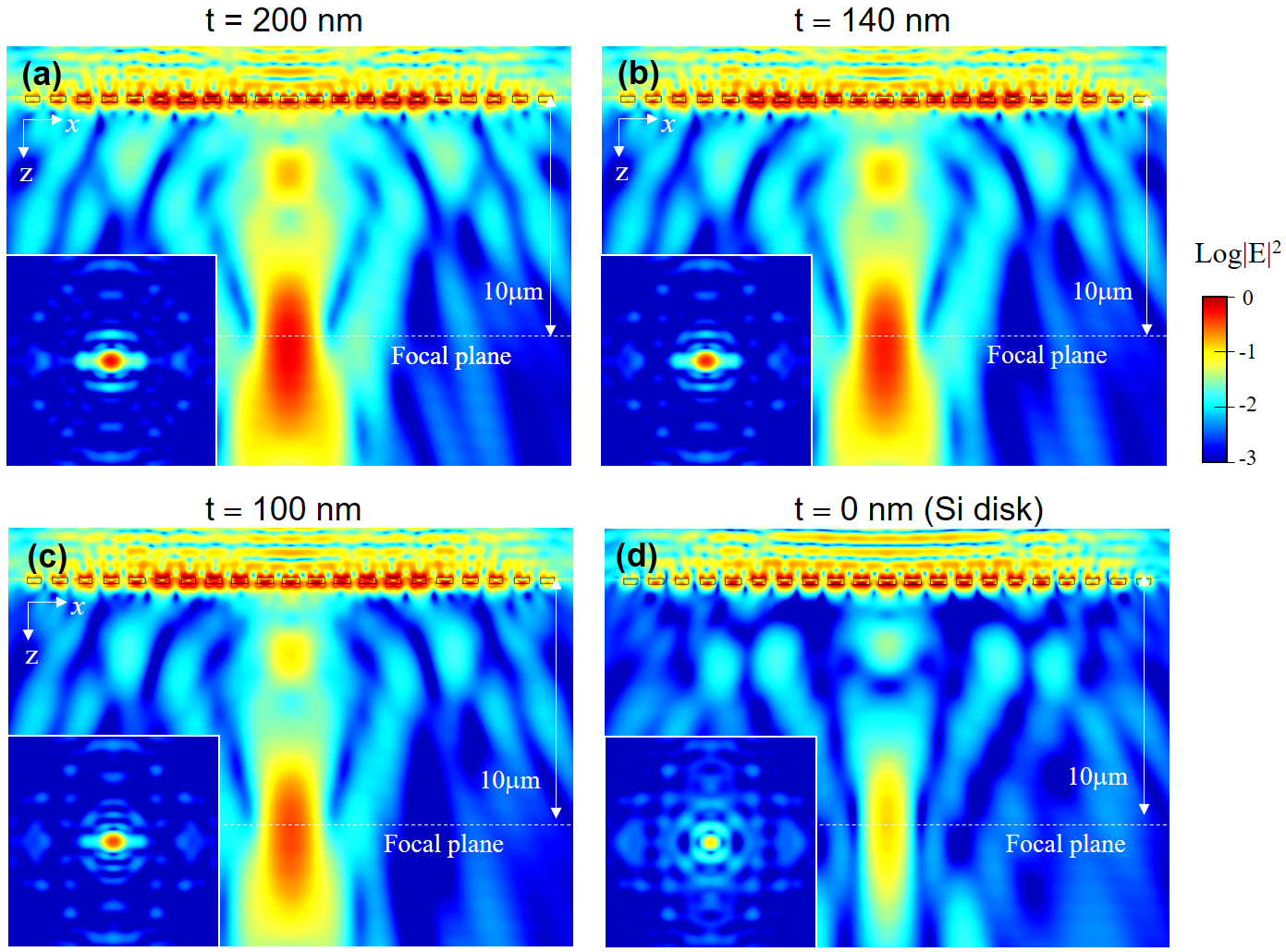


Fig. S7 Intensity distribution for the Si/ma-N metalens with (a) 200-nm-thick ma-N layer, (b) 140-nm-thick ma-N layer, (c) 100-nm-thickn ma-N layer, and (d) without ma-N layer. Here, we set as the focal length of the metalens, *f* = 10 μm, and the wavelength of incident light, λ = 1500 nm. All the images are *xz*-plane views along the optical axis and the inset images are *xy*-plane views at the focal plane.


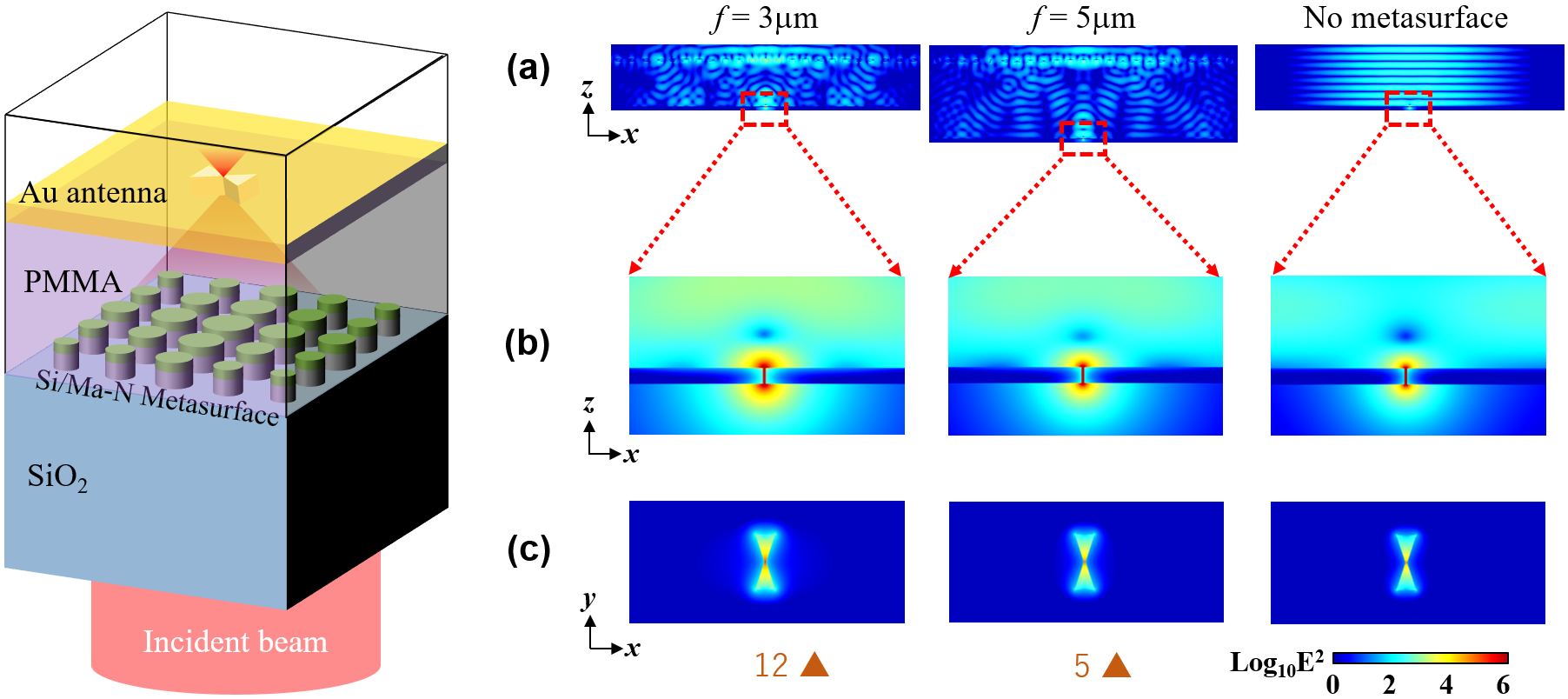


Fig. S8: Numerical results of the surface plasmon resonance sensor (SPR) composed of Si/ma-N metalenses with various focal lengths. (left) illustration of the SPR sensor. (a) Intensity distributions of the focusing beam by the Si/ma-N metalenses with various focal lengths and without the metalens on a logarithmic scale. (b) *Magnified* view of the intensity distribution near the Au bowtie air hole in the *xz*-plane and (c) *xy*-plane.
